# Supplementary material for: Gut Microbiota in Patients with Non-Alcoholic Fatty Liver Disease without Type 2 Diabetes: Stratified by Body Mass Index
Source: Int J Mol Sci. 2024 Feb 2;25(3):1807. doi: 10.3390/ijms25031807 (PMC10855659; doi:10.3390/ijms25031807)
Supplement: Supplementary file 1 [file ijms-25-01807-s001.zip › Supplementary Materials Figures and Table.pdf]

Supplementary files

Gut microbiota in patients with non-alcoholic fatty liver disease without type 2 diabetes: Stratified by body mass index

Natthaya Chuaypen [1,2], Aisawan Asumpinawong [3], Pattarose Sawangsri [3], Jakkrit Khamjerm [1,4], Nutta Iadsee [1], Thananya Jinato [1], Sawannee Sutheeworapong [6], Suthep Udomsawaengsup [3] and Pisit Tangkijvanich [1,*]

1 Center of Excellence in Hepatitis and Liver Cancer, Department of Biochemistry, Faculty of Medicine, Chulalongkorn University, Bangkok 10330, Thailand; natthaya.ch56@gmail.com (N.C.); jakkrit.champer@gmail.com (J.K.); nutta.i@kkumail.com (N.I.); ji.thananya@gmail.com (T.J.)
2 Metabolic Diseases in Gut and Urinary System Research Unit (MeDGURU), Department of Biochemistry, Faculty of Medicine, Chulalongkorn University, Bangkok 10330, Thailand
3 Treatment of Obesity and Metabolic Disease Research Unit, Department of Surgery, Faculty of Medicine, Chulalongkorn University, Bangkok 10330, Thailand; aisawan.asum@gmail.com (A.A.); spattarose@gmail.com (P.S.); suthep.u@chula.ac.th (S.U.)
4 Biomedical Engineering Program, Faculty of Engineering, Chulalongkorn University, Bangkok 10330, Thailand
5 Medical Biochemistry Program, Faculty of Medicine, Chulalongkorn University, Bangkok 10330, Thailand
6 Systems Biology and Bioinformatics Research Unit, Pilot Plant Development and Training Institute, King Mongkut's University of Technology Thonburi, Bangkok 10150, Thailand; s.sawannee@gmail.com
* Correspondence: pisittkvn@yahoo.com

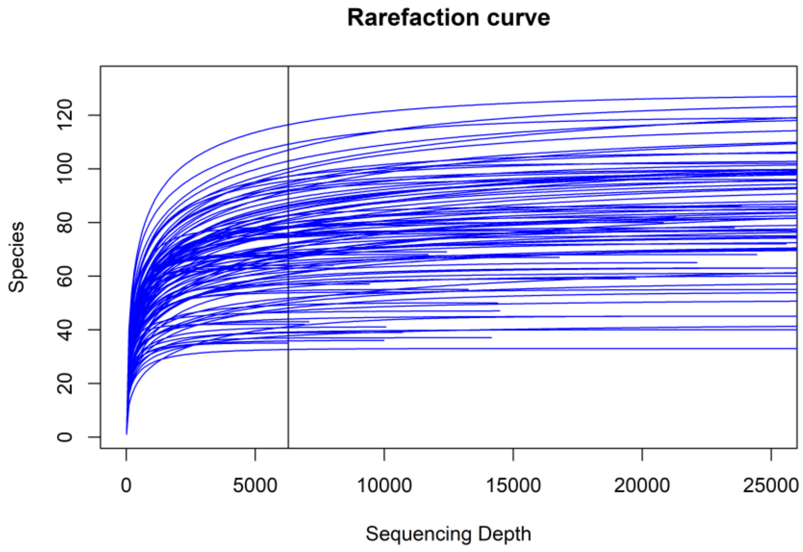

Figure S1. Rarefaction curve.

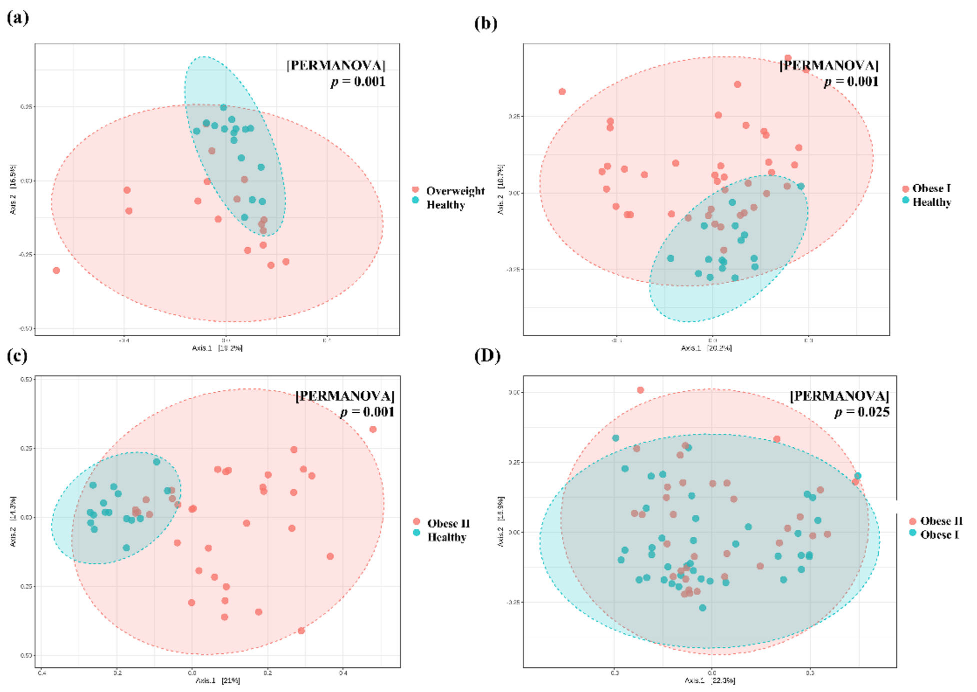

Figure S2. Subgroup analysis of bacterial community based on Bray-Curtis dissimilarity.

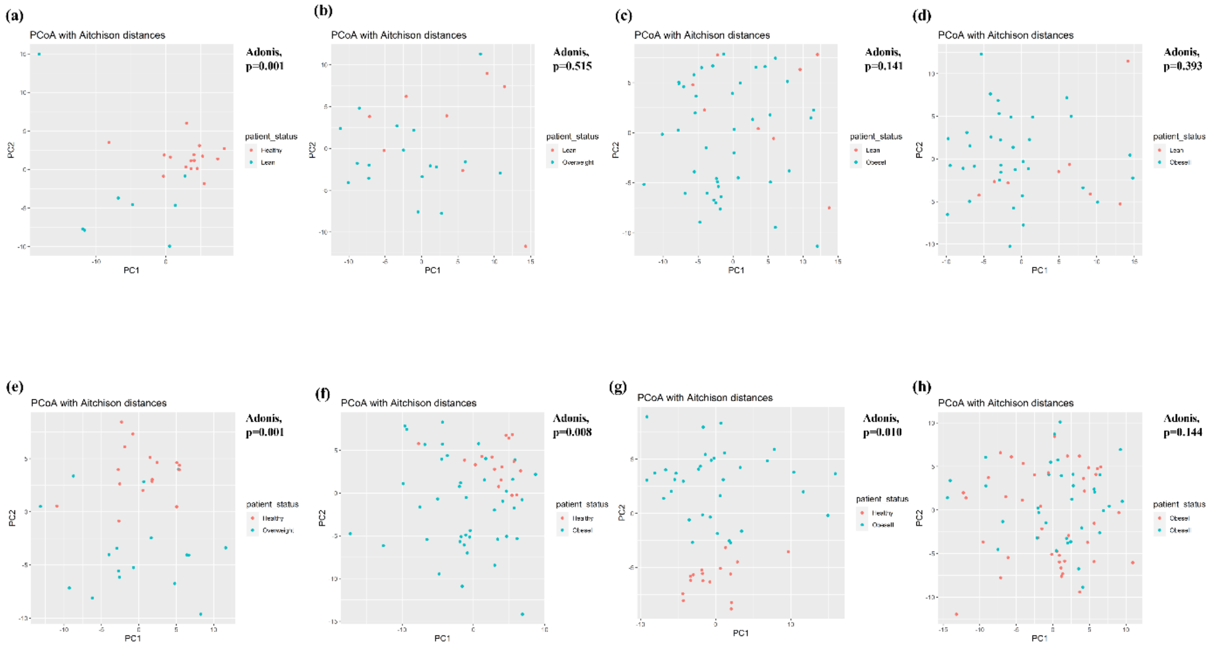

Figure S3. PCoA plot based on Aitchison distance.

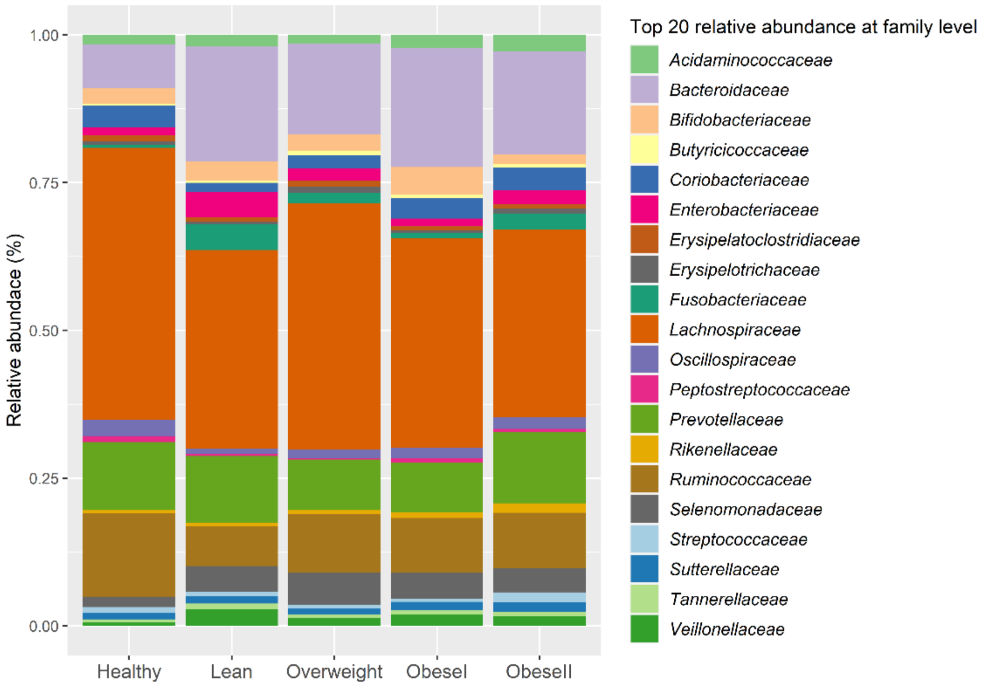

Figure S4. Relative abundance of bacteria at family level.

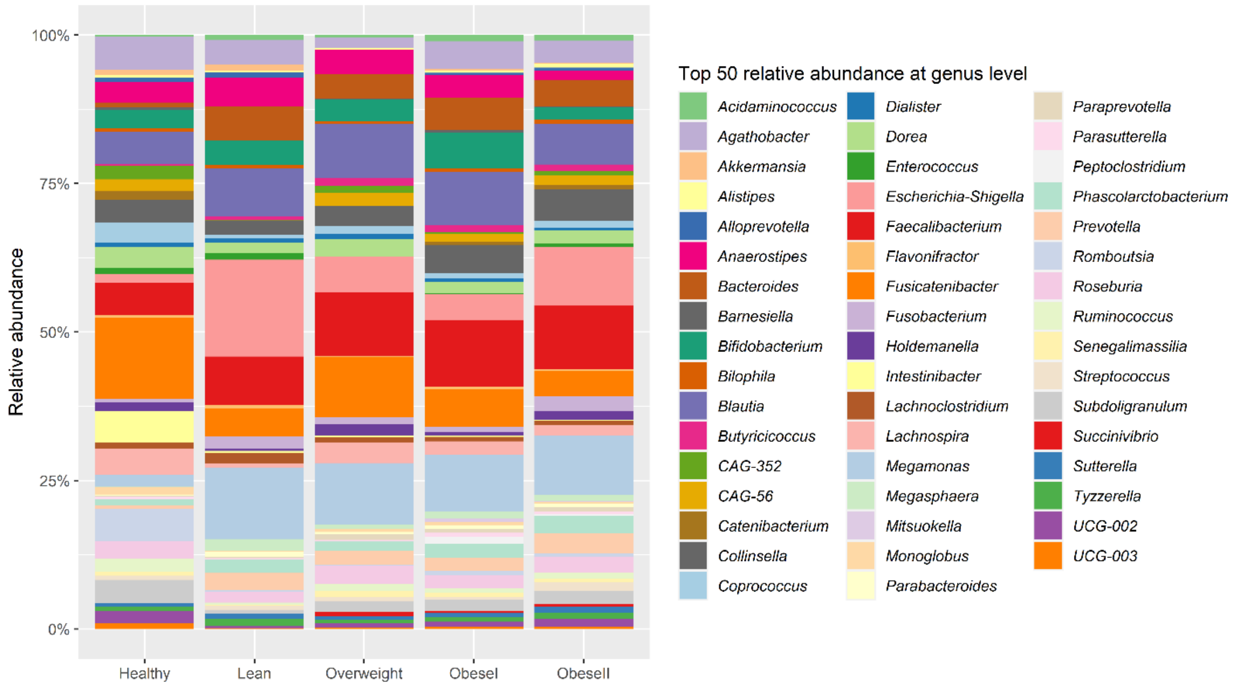

Figure S5. Relative abundance of bacteria at genus level.

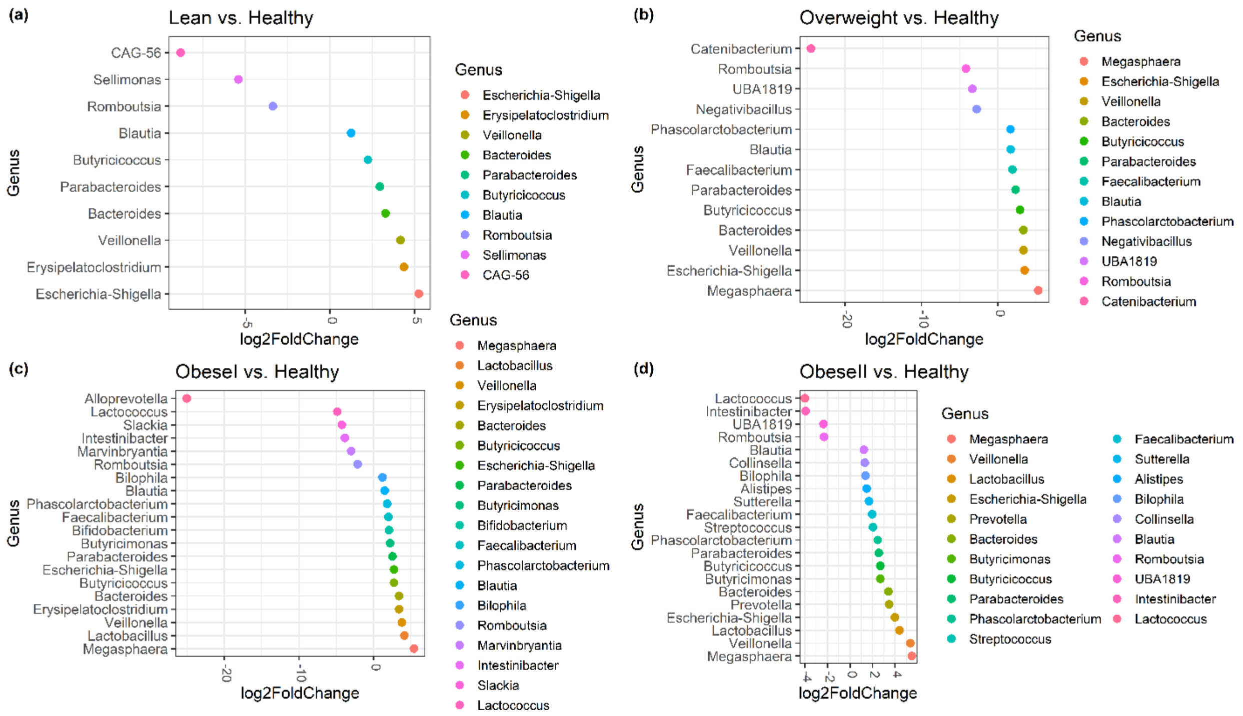

Figure S6. Differential bacteria in healthy control vs lean (**a**) and non-lean subgroups analysis (**b**-**d**) using DESeq2.

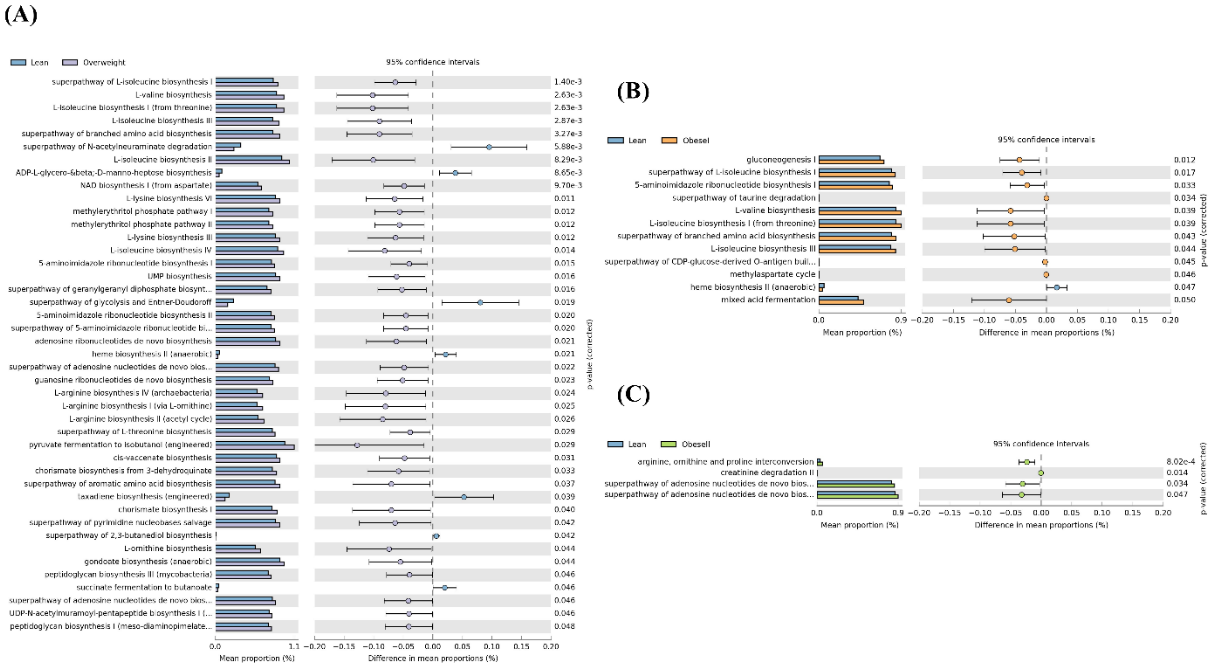

Figure S7. The functional pathways associated with ranges of BMI in subgroup NAFLD. (a) lean vs. overweight, (b) lean vs. obese I, (c) lean vs. obese II.

Table S1. 16S data processing read counts.

Sample ID	input	filtered	denoisedF	denoisedR	merged	nonchim
H33	145822	119400	90060	117765	69770	35204
H43	125292	100601	79307	99808	64507	50012
H34	111503	87645	71103	86300	52586	33234
H44	124626	97746	69619	96822	58122	36397
H35	85995	68960	50393	68653	46394	42562
H45	131374	104608	85075	103003	63229	29637
H43	90694	74836	54853	73833	42116	29261
H36	121563	95885	70322	95156	58540	35106
H46	115499	94572	70137	93939	58623	43666
H37	102281	82639	71403	80602	52536	35277
H47	113091	89691	76481	87819	56202	32523
H48	111019	85878	65047	85144	53708	40046
H39	102335	81557	64634	79831	47072	31352
H40	117615	91965	71912	90390	55833	43109
H41	111842	86911	66275	85821	53929	45505
H42	99818	81854	59878	81303	53231	47091
F_FF66_1	78897	65434	64865	64900	64060	63107
F_FF66_100	59485	25707	25489	25379	24590	24435
F_FF66_101	50787	41925	41457	41538	40914	40742
F_FF66_103	38115	17239	17002	16944	16433	16146
F_FF66_106	104749	88642	87866	88042	86219	84211
F_FF66_107	73983	62208	61631	61751	60475	59485
F_FF66_108	48567	20341	20201	20156	19911	19749
F_FF66_11	59434	48056	47699	47717	47217	47137
F_FF66_110	23743	10515	10426	10383	9987	9977
F_FF66_112	34023	28823	28632	28623	28257	28081
F_FF66_113	17586	7469	7371	7308	7074	7074
F_FF66_114	142003	112017	111209	111164	109480	108103
F_FF66_115	73976	63576	62890	62998	62105	61425
F_FF66_117	18860	8737	8561	8502	8108	8020
F_FF66_118	19779	8978	8872	8863	8623	8619
F_FF66_12	56577	45953	45523	45508	44807	44607
F_FF66_120	93971	81786	81125	81140	79799	76682
F_FF66_121	33606	14685	14611	14567	14183	14146
F_FF66_123	30156	24538	24261	24212	23844	23842
F_FF66_124	42962	34831	34575	34603	34114	34020
F_FF66_126	32709	26357	26159	26133	25688	25567
F_FF66_128	59234	27911	27756	27715	27344	26615
F_FF66_129	44450	16454	16274	16249	15834	15810
F_FF66_130	23759	10087	9982	9968	9589	9421

F_FF66_131	33849	15305	15150	15148	14523	14473
F_FF66_134	123778	107711	106739	106993	104900	103130
F_FF66_136	23144	11052	10853	10788	10191	10058
F_FF66_137	56433	24635	24366	24298	23869	23570
F_FF66_138	26950	12886	12740	12706	12410	12408
F_FF66_140	67397	32358	32170	32092	31454	31181
F_FF66_141	22160	10966	10900	10863	10688	10688
F_FF66_144	297902	118740	117929	118099	115928	114607
F_FF66_145	92432	80783	80222	80173	79093	77800
F_FF66_146	37848	32005	31224	31239	30157	29640
F_FF66_147	66333	56995	56526	56536	55676	54831
F_FF66_149	49278	21926	21630	21564	21001	20817
F_FF66_150	94212	82695	82014	82106	80787	79541
F_FF66_152	29535	14664	14339	14419	13533	13246
F_FF66_155	51510	22795	22627	22556	22230	22112
F_FF66_156	25232	12277	12097	12068	11653	11653
F_FF66_157	16902	7826	7719	7704	7459	7459
F_FF66_16	127397	106536	106014	105902	104742	103705
F_FF66_161	49886	43467	43192	43148	42565	42311
F_FF66_162	67031	55447	55080	55073	54114	53999
F_FF66_163	58595	50368	50096	50104	49317	49006
F_FF66_164	38847	34073	33857	33813	33351	33175
F_FF66_167	60207	54605	54429	54440	53974	53311
F_FF66_169	46086	39268	38979	39012	38529	38217
F_FF66_17	28004	11221	11096	11059	10845	10832
F_FF66_23	49518	40839	40557	40532	40130	39928
F_FF66_25	51889	41000	40664	40733	40009	39848
F_FF66_28	128918	100715	100182	100160	98449	97275
F_FF66_36	58167	48051	47760	47739	47361	47128
F_FF66_38	31333	12398	12249	12209	11716	11697
F_FF66_42	113727	91301	90695	90719	89614	88051
F_FF66_44	337822	264713	263169	263156	259981	257380
F_FF66_46	130437	102433	102116	102074	99973	98558
F_FF66_48	24735	19092	18894	18858	18433	18427
F_FF66_5	19761	8543	8381	8370	8103	8098
F_FF66_50	62877	50638	50232	50304	49720	49030
F_FF66_51	43357	34458	34145	34168	33518	33446
F_FF66_53	46124	35424	35153	35127	34703	34636
F_FF66_54	16125	7488	7376	7351	7206	7206
F_FF66_55	88183	68674	68157	68073	67464	67147
F_FF66_56	83247	66612	66030	66030	65324	65102
F_FF66_57	238908	193812	192710	192934	189973	187006
F_FF66_58	22123	7624	7487	7417	6904	6904

F_FF66_6	96716	79272	78723	78781	77655	77035
F_FF66_62	21016	17176	16976	16997	16784	16777
F_FF66_66	45126	36702	36283	36412	35936	35824
F_FF66_67	49707	21982	21750	21669	20883	20871
F_FF66_68	34856	28370	28135	28150	27808	27565
F_FF66_70	62227	49610	49205	49192	48513	48392
F_FF66_71	48660	38955	38666	38682	38173	37962
F_FF66_82	58064	45855	45547	45537	44652	44441
F_FF66_83	75400	62330	61829	61877	61078	60528
F_FF66_84	63085	50313	49988	49996	49393	48827
F_FF66_85	113721	94872	94380	94399	93261	90774
F_FF66_86	228248	186913	186081	186131	184013	180154
F_FF66_89	45443	38801	38385	38479	37753	37643
F_FF66_9	33098	26919	26602	26640	26260	26121
F_FF66_91	19874	6511	6436	6425	6275	6275
F_FF66_93	32949	25003	24839	24804	24319	23926
F_FF66_96	38229	31380	31207	31207	30880	30732
F_FF66_97	35224	14735	14633	14587	14406	14386
F_FF66_98	34298	27176	26885	26939	26590	26524
GO_B01	63330	56821	56528	56548	55746	55435
GO_B02	40893	35531	35255	35288	34876	34758
GO_B03	64805	55918	55596	55595	55001	54695
GO_B04	46670	40132	39846	39868	39222	38885
GO_B05	60448	52061	51734	51762	51147	50920
GO_B07	45244	37598	37351	37387	37022	36823
GO_B08	59419	51572	51187	51304	50390	50237
GO_B09	55450	49094	48791	48832	48236	47712
GO_B10	72959	60451	60121	60165	59417	58762
GO_B11	70846	59669	59176	59204	58250	58041
GO_B12	31064	26237	25935	25958	25582	25531
GO_B15	24821	21637	21486	21495	21304	21284
GO_B16	42799	37314	37085	37071	36532	36304
GO_B17	35755	30433	30231	30244	29908	29760
